# A shot in the dark: Comparative morphology of the bioluminescent tube organs in tubeshoulders (Platytroctidae)

**DOI:** 10.1371/journal.pone.0332016

**Published:** 2026-01-14

**Authors:** Emily M. Carr, Rene P. Martin, Michael J. Ghedotti, John S. Sparks

**Affiliations:** 1 American Museum of Natural History, Department of Ichthyology, Division of Vertebrate Zoology, New York, New York, United States of America; 2 Richard Gilder Graduate School, American Museum of Natural History, New York, New York, United States of America; 3 University of Nebraska-Lincoln, School of Natural Resources, Lincoln, Nebraska, United States of America; 4 Regis University, Department of Biology, Denver, Colorado, United States of America; CONICET: Consejo Nacional de Investigaciones Cientificas y Tecnicas, ARGENTINA

## Abstract

Bioluminescence, visible light produced by a living organism, is a key innovation in the diversification of deep-sea fishes. It is useful for a myriad of behaviors and interactions in deep-sea organisms, including communication, predation, camouflage via counterillumination, and predator avoidance. In this study, we investigate the deep-sea tubeshoulders (Platytroctidae), fishes that possess a unique postcleithral tube organ associated with their shoulder girdle that excretes bioluminescent fluid, a feature that unites all members of this poorly studied family. Many tubeshoulders also possess additional bioluminescent structures and luminescent tissues, including a series of tube organs on the caudal peduncle unique to *Platytroctes apus* that are hypothesized to be similar in structure and function to the postcleithral tube organ. Herein, we present the first histological analysis of the caudal tube organs in *P. apus* and use histological methods to investigate the morphological diversity in postcleithral tube organ structure across 14 platytroctid species, representing 10 of 13 valid genera. We show that the postcleithral tube organ generally exhibits a conserved morphology across genera and species. However, several species-specific anatomical differences are noted. In some individuals, we observe the presence of luminescent-fluid cells within the tube organ in various stages of development, which may provide evidence for inferring the type of secretory gland found in this novel bioluminescent light organ. We also show that the structure of the caudal tube organs in *P. apus* are similar to the postcleithral tube organ present in all members of Platytroctidae, likely indicating a similar luminescent fluid emission function.

## Introduction

Bioluminescence is the production of visible light by a living organism via a chemical reaction between luciferin, a light producing molecule, and luciferase, an enzyme [1–3]. Bioluminescence is phylogenetically widespread and has evolved numerous times across the tree of life [4,5]. This functionally diverse phenomenon is hypothesized to be used in predator avoidance, prey capture, mating behaviors, and camouflage via counterillumination [6–9]. The numerous functions of marine bioluminescence are facilitated by both a diversity of light organ structures and photogenic tissues, as well as numerous mechanisms for producing and emitting light. Bioluminescence in marine fishes is generally achieved via one of two ways, either by housing symbiotic bioluminescent bacteria (e.g., *Allivibrio*, *Photobacterium*, *Vibrio*) in internal structures, or producing luciferase intrinsically and obtaining the substrate luciferin from their diets [1,5,6,8,10,11]. Bioluminescent fishes also exhibit considerable variation in their light emitting structures, which can range from simple patches of bioluminescent tissue [7] to significantly more anatomically complex structures, including internal light organs, as well as light emitting photophores, chin barbels, and lures [8,12–14]. Many light organs also include associated structures to control the direction and emission of light [12,15–18].

However, one type of bioluminescence that is considerably less common in fishes involves structures that emit, eject, or secrete bioluminescent fluid into the surrounding environment [19,20]. Secretory luminescence has independently evolved in numerous marine lineages of invertebrates and vertebrates, including annelids, cephalopods, ostracods, copepods, mysid and decapod shrimps, elasmobranchs, and teleosts [19]. Secretory luminescence is generally hypothesized to function in predator avoidance by creating a distraction or “smoke screen”, allowing light-emitting organisms to escape [21,22]. However, luminescent secretions may also be used for sexual displays or marking potential predators with light [23–25]. Interestingly, most species with luminescent secretions produce their bioluminescence endogenously, and not from symbioses with luminescent bacteria [19]. Although the function of secretory luminescence is hypothesized to be relatively conserved across lineages, both the location of these light organs and the cellular mechanisms facilitating the production of the secretion are highly variable [21,25–30]. In vertebrates, luminous secretions are limited to two genera of marine elasmobranch (*Mollisquama and Euprotomicroides*) [19,31], and a single family of bony fishes (Platytroctidae) [32]. In the American Pocket Shark (*Mollisquama mississippiensis*) the light organ is comprised of pectoral pockets and is hypothesized to secrete luminescent fluid via epidermal holocrine glands, whereby expulsion is triggered via movement of the pectoral fin [19]. In the taillight shark (*Euprotomicroides zantedeschia*) luminescent fluid is hypothesized to be emitted from a pelvic pouch [32]. However, the mechanism of secretory luminescence remains poorly understood in Platytroctidae.

**Fig 1:**
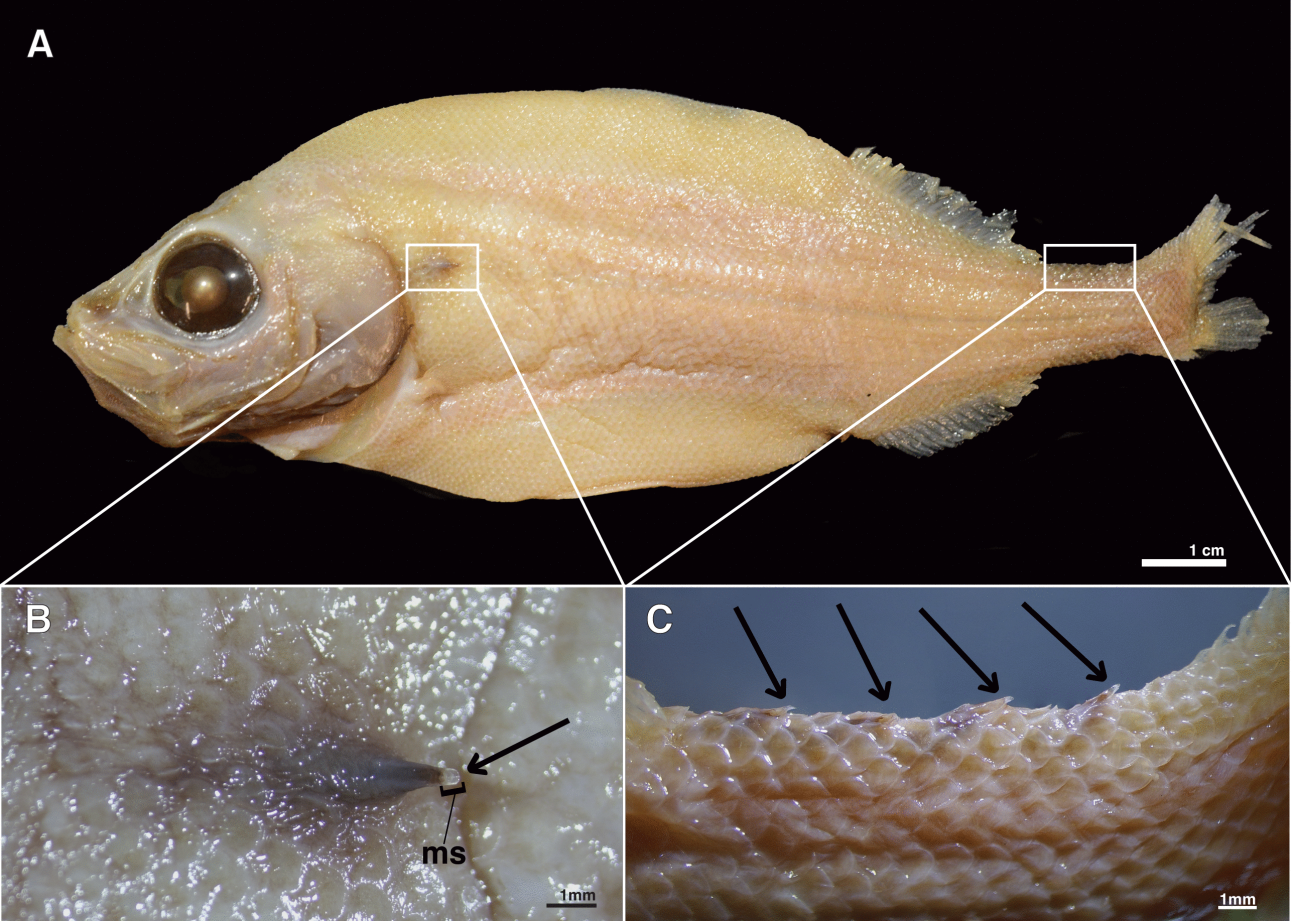
External anatomy of the postcleithral and caudal tube organs (A) *Platytroctes apus* (SIO 55-244, 136.4 mm SL) showing location of postcleithral tube organ (left white box) and caudal tube organs (right white box). **(B)** Postcleithral tube organ in *P. apus* (SIO 77-54) showing terminal opening and modified scale (ms). **(C)** Caudal tube organs on dorsal margin of caudal peduncle in *P. apus* (SIO 55-244). Arrows indicate the terminal opening of the postcleithral and caudal tube organs.

Platytroctidae is a morphologically diverse family comprising 40 species arrayed within 13 genera of globally distributed (except the Mediterranean Sea) deep-sea fishes. Platytroctids are found in mesopelagic to bathypelagic depths (200–4000 m) [32]. Mesopelagic genera include *Persparsia, Sagamichthys, Searsia, and Holtbyrnia,* while the remaining nine genera are found at deeper bathypelagic depths [32]. Platytroctids are zooplanktivores, with a diet consisting of small crustaceans, chaetognaths, and cnidarians [33]. Members of the family, commonly called tubeshoulders, are united by the presence of an apomorphic postcleithral tube organ, a unique sac-like internal structure situated beneath the cleithrum that can emit bioluminescent fluid into the surrounding environment via an external tube [32] (Fig 1). This strategy is hypothesized to be used for the same function as other secretory luminescent taxa, either to escape predation or ward off predators [32]. As a result, members of this family have been informally referred to as “luminous cloud-throwers” [34]. Whereas luminescent fluid emission in platytroctids has been mentioned previously in the literature [32,34–36], only one photograph has been published to date in a peer-reviewed study [20]. Many platytroctids also possess additional discrete light organs embedded in ventral regions of their epidermis  thought to be used in counterillumination [20], as well as morphologically distinct patches of luminescent tissue on several different body regions that vary considerably among genera. One species, *Platytroctes apus,* has an additional series of openings on the dorsal and ventral margins of the caudal peduncle leading to darkly pigmented chambers, although these structures have not been studied in detail [32].

Beebe [35] documented the reduction of the external projection of the postcleithral tube organ over ontogeny and described a modified scale that supports the tube in one platytroctid species, *Mentodus rostratus* [35]. Histological analyses of the tube organ are limited to a single study that examined only one species, *Sagamichthys schnakenbecki* [22], and we know very little regarding the composition of the bioluminescent fluid of the tube organ or its glandular basis. Nicol [22] observed three distinct cell types in the darkly pigmented lumen of tube organ, and hypothesized they represented three developmental stages of the luminescent fluid cells. However, Nicol [22] did not find strong evidence of the gland type, suggesting that the tube organ may be a unique type of exocrine gland. Overall, the postcleithral tube organ in platytroctids remains extremely understudied and virtually no variation in this organ has yet been documented among platytroctid species. In addition, the internal structure and function of the caudal tube-like organs present only in *P. apus* remain unknown.

In this study we describe the morphology and ultrastructure of the postcleithral tube organ across Platytroctidae using histological techniques, and document and formally describe similar tube-like organs that occur on the upper and lower margins of the caudal peduncle in *Platytroctes apus*. This study is critical in advancing our knowledge of platytroctid tube organs as a luminescence ejection system, an extremely rare phenomenon in fishes. We present a taxonomically comprehensive comparative analysis of the tube organ across 10 of 13 recognized platytroctid genera. We also provide the first histological sections and detailed description of the structure of the caudal tube organs that are unique to *P. apus*.

## Methods

All specimens sectioned for histological analysis were formalin-fixed and alcohol-preserved (75% ethanol or 50% isopropanol) museum specimens from multiple institutions (Table 1). No animal research ethics committee approval was required for this research. Gross anatomy of the tube organs (e.g., the presence of modified scales) was examined using a Nikon SMZ800N stereo microscope. The tube organ (right side) and surrounding tissue was dissected for histological preparation in 14 platytroctid species representing 10 of the 13 total genera (Fig 1B; Table 1). Two of the dorsal caudal tube organs were also dissected for histological sectioning in *Platytroctes apus* (n = 1; Fig 1C). Presence of the caudal tube organ was determined externally by examining the caudal peduncle of all species using a Nikon SMZ800N stereo microscope. Many platytroctid species are rarely collected, and as a result, are poorly represented in museum collections. Thus, dissections of additional individuals and several species were not possible due to restrictions of the loaning institution regarding destructive sampling. Specimens examined for the presence of caudal tube organs in addition to specimens used for histological analyses (Table 1) are listed in the supplementary materials (S1 Fig).

**Table 1:** Platytroctid species examined for histological analysis of postcleithral and caudal tube organ structure in this study.

Genus	Species	Catalog number	Standard Length (mm)	Modified Scale in Postcleithral Tube Organ	Caudal Tube Organ*
*Barbantus*	*curvifrons*	MCZ 60746	77.2	Present	Absent
*Holtbyrnia*	*latifrons*	SIO 80-110	174.4	Present	Absent
	*macrops*	LACM 30432-26	21.9	Absent	Absent
	*melanocephala*	LACM 31065-9	34.9	Present	Absent
*Maulisia*	*microlepis*	MCZ 168334	121.6	Present	Absent
*Mentodus*	*facilis*	LACM 9706-42	46.2	Present	Absent
*Mirorictus*	*taningi*	SIO 14-136	118.1	Present	Absent
*Normichthys*	*operosus*	MCZ 163242	129.7	Present	Absent
	*yahganorum*	SIO 61-45	42.8	Present	Absent
*Platytroctes*	*apus*	SIO 77-54	130.2	Present	Present
	*mirus*	MCZ 138114	101.8	Present	Absent
*Sagamichthys*	*abei*	SIO 18-4	209.2	Present	Absent
*Searsia*	*koefoedi*	MCZ 168583	137.3	Present	Absent
*Searsioides*	*multispinus*	SIO 61-32	101.9	Present	Absent

*The presence of the caudal tube organ was determined externally by examining the caudal peduncle of all species using a Nikon SMZ800N stereo microscope. Each catalog number represents one individual.

Tissues were dehydrated through a stepwise series of increasing concentrations of EtOH to 100% and incubated in xylene. Tissues were subsequently embedded in paraffin wax and sectioned laterally through the tube organ at 5–10 μm intervals using a Leica HistoCore MULTICUT rotary microtome. Sections were mounted on charged slides with distilled water and dried at 50°C for 24 hours. Slides were then stained with Mason’s Trichrome following the protocol from Ghedotti et al. [14]. Stained sections were mounted in Canada balsam and imaged using a Nikon Eclipse 50*i* compound microscope equipped with an Excelis 4K UHD camera. Images used in figures were prepared by increasing brightness, eliminating erroneous debris caused by the mounting process, and by combining photographs into composite images using Adobe Photoshop. Institutional abbreviations of specimens used for histological analysis in this study are: Natural History Museum of Los Angeles County (LACM), Museum of Comparative Zoology, Harvard University (MCZ), and Scripps Institution of Oceanography, University of California, San Diego (SIO).

## Results

### Postcleithral tube organ

**Fig 2:**
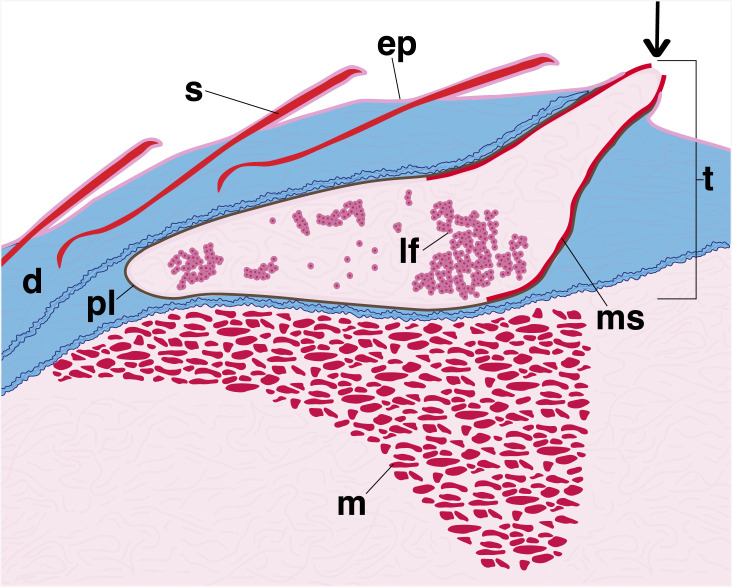
Generalized illustration showing internal postcleithral tube organ anatomy. Abbreviations: scale (s, red), epithelium (ep, dark pink), dermis (d, blue), tube organ **(t)**, pigment layer (pl, brown), modified scale (ms, red), luminescent fluid (lf, pink), muscle (m, red). Arrow indicates terminal tube opening, which is directed posteriorly.

**Fig 3:**
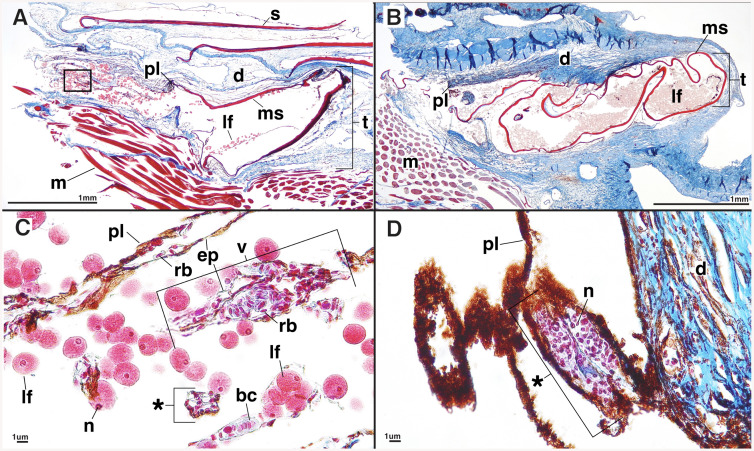
Longitudinal sections through the postcleithral tube organ. **(A)** *Mirorictus taningi* (SIO 14-136) and **(B)** *Searsia koefoedi* (MCZ 168583). Potential developing luminescent-fluid cells (*) in **(C)** *Mirorictus taningi* (SIO 14-136) and **(D)** *Platytroctes apus* (SIO 77-54). Anterior is to the left in all figure panels, such that the posteriorly-directed terminal opening of the tube organ is to the right. Abbreviations: scale **(s)**, dermis **(d)**, tube organ **(t)**, pigment layer (pl), modified scale (ms), luminescent fluid (lf), muscle **(m)**, nucleus **(n)**, blue cells (bc), red blood cells (rb), blood vessel **(v)**, epithelium (ep).

**Fig 4:**
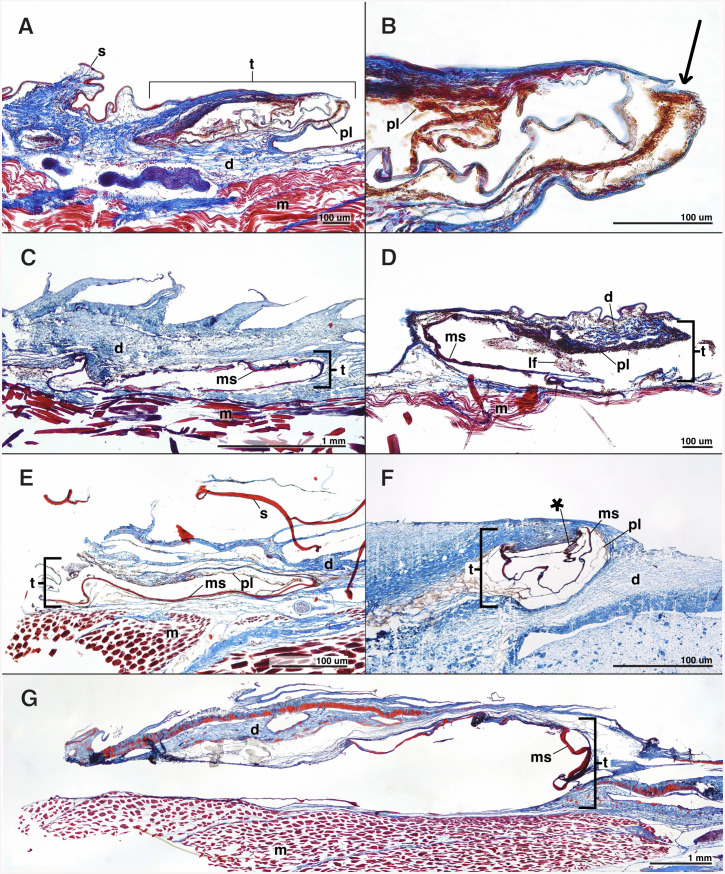
Longitudinal sections of the postcleithral tube organ. **(A and B)** *Normichthys yahganorum* (SIO 61-45), **(C)** *Normichthys operosus* (MCZ 163242), **(D)** *Holtbyrnia melanocephala* (LACM 31065-9), **(E)** *Mentodus facilis* (LACM 9706-42), **(F)** *Platytroctes apus* (SIO 77-54), and **(G)** *Sagamichthys abei* (SIO 18-4). Anterior is to the left in all figure panels, such that the posteriorly-directed terminal opening of the tube organ is to the right. Abbreviations: scale **(s)**, dermis **(d)**, tube organ **(t)**, pigment layer **(pl)**, modified scale **(ms)**, luminescent fluid **(lf)**, muscle **(m)**, blood vessel **(v)**, potential developing luminescent-fluid cells (*). Arrow in panel B points towards terminal tube opening.

We observed overall similar morphology in the postcleithral tube organ across all species examined, including *Barbantus curvifrons, Holtbyrnia latifrons, H. macrops, H. melanocephala, Maulisia microlepis, Mentodus facilis, Mirorictus taningi, Normichthys operosus, N. yahganorum,* *Platytroctes apus, P. mirus, Sagamichthys abei, Searsia koefoedi,* and *Searsioides multispinus* (Figs 2–4, Table 1, S2 Fig). The following description is applicable to the anatomy of all species, and any exceptions are noted. The postcleithral tube organ is located at or ventral to the lateral line, posterior to the opercular series, and ventroposterior to the cleithrum (Fig 1A). The external tip (= opening) of the tube is oriented posteriorly and is associated with a modified scale (Fig 1B) in all species examined except for *H. macrops* (Figs 1, 3, 4, S2 Fig). When present, this modified scale (ms) is curved into a conical shape, extends internally (i.e., inside the lumen of the tube for about 1/3–1/2 of its length; Fig 2), and supports the external opening of the tube organ (Figs 2–4). The tube organ (t) lies within the dermis (d) below the scales (s) and epidermis in all specimens examined. The lumen of the usually scale-supported tube extends anteroventrally from the external opening (tip) of the organ and is located adjacent and superficial to the hypaxial musculature (m) of the body wall. Within the tube organ, a dense pigment layer (pl) of melanophores lines the wall of the lumen, becoming denser apically, nearer the opening to the external environment (Fig 2).

The lumen of the postcleithral tube organ contains spherical luminescent-fluid cells (lf) in the following species: *B.* curvifrons, *H. latifrons, H. melanocephala, M. taningi, P. apus, P. mirus,* and *S. koefoedi* (Figs 2–4, S2 Fig). These cells have darkly stained nuclei with surrounding granules that stain red (Fig 3A and C). In two species, *S. koefoedi* and *P. mirus,* the granules of the luminescent-fluid cells are lightly stained, and the nucleus is clear, likely an artifact of poor stain uptake (Fig 3B, S2 Fig). The diameter of the luminescent-fluid cells ranges from 8–32 μm, with an average diameter of 18.4 μm (Table 2, S1 Table). In all species except *M. microlepis* and *N. operosus*, smaller cells are also present within the dermis surrounding the lumen of the tube organ (*) (Fig 3C-D, S2 Fig), often associated with the pigment layer (pl). These cells have very limited cytoplasm that is lightly stained relative to the fully developed luminescent-fluid cells (lf), however, the nuclei remain darkly stained (Fig 3C-D, S2 Fig). Although the size of these cell clumps (*) (Fig 3C-D) varies both among species and within specimens, the component cells have a consistent diameter of 2–7 μm, with an average cell size of 4.6 μm (Table 2, S1 Table). A third cell type, blue cells (bc), are also found in the lumen of the tube in *B. curvifrons, H. latifrons, H. macrops, M. facilis, M. taningi, N. yahganorum, P. mirus,* and *S. koefoedi* (Fig 3C, S2 Fig). Blue cells (bc) have a darkly stained nucleus and an unstained or lightly blue stained cytoplasm, and range in diameter from 6–13 μm (Fig 3C, Table 2, S1 Table). The postcleithral tube organ is also highly vascularized, and we observed the presence of numerous oblong red blood cells in all species examined (rb; 5–12 μm) that are contained within blood vessels (v; Fig 3C, Table 2, S1 Table).

**Table 2:** Ranges in diameter of luminescent fluid cells, blue cells, potential developing luminescent fluid cells, and blood cells.

Genus	Species	Luminescent Fluid Cells (μm)	Blue Cells (μm)	Developing Luminescent Fluid Cells (μm)	Blood Cells (μm)
*Barbantus*	*curvifrons*	12-28	7-12	3-5	5-10
*Holtbyrnia*	*latifrons*	12-23	9-13	4-7	7-9
	*macrops*	—	6-12	2-4	7-10
	*melanocephala*	8-19	—	3-5	7-12
*Maulisia*	*microlepis*	—	—	—	5-8
*Mentodus*	*facilis*	—	6-10	4-5	6-10
*Mirorictus*	*taningi*	13-25	8-10	3-6	8-12
*Normichthys*	*operosus*	—	—	—	6-12
	*yahganorum*	—	6-7	4-7	5-11
*Platytroctes*	*apus*	21-26	—	3-7	5-10
	*mirus*	12-28	11-13	3-6	7-12
*Sagamichthys*	*abei*	—	—	4-7	5-7
*Searsia*	*koefoedi*	14-32	10-11	2-6	6-12
*Searsioides*	*multispinus*	*	Present*	*	*

Measurements were taken on a Nikon Eclipse 50*i* compound microscope equipped with a calibrated Excelis 4K UHD camera. All measurements can be found in the supplementary information (S1 Table). An em dash (—) signifies that a certain cell type was not observed in the specimen examined. *Additional observations or measurements of these cell types could not be conducted due to slide damage.

### Caudal peduncle tube organs

**Fig 5:**
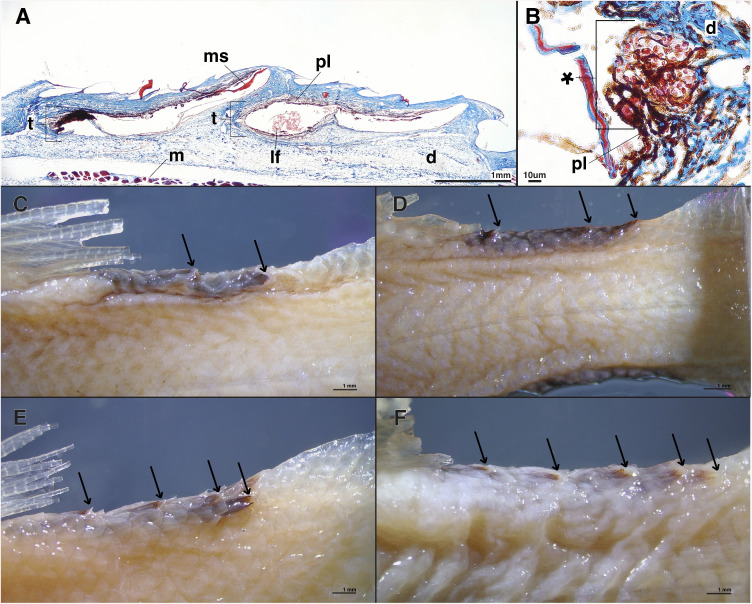
Longitudinal histological sections and external anatomy of the caudal tube organ in *P. apus* (SIO 77-54). **(A)** Two dorsal caudal tube organs in lateral view and **(B)** potential developing luminescent-fluid cells (*) in the lumen of a caudal tube organ in *P. apus*. Variation in the number of dorsal caudal tube organs in *Platytroctes apus*, showing **(C)** two (USNM 206894), **(D)** three (USNM 206894), **(E)** four (USNM 206893), and **(F)** five (USNM 201649) caudal tube organs. Arrows point towards terminal caudal tube opening. Note: Ventral tube organs on the caudal peduncle in *P. apus* also vary in number. Anterior is to the left in all figure panels, such that the posteriorly-directed terminal openings of the caudal tube organs are located to the right. Abbreviations: dermis **(d)**, tube organ **(t)**, pigment layer **(pl)**, modified scale **(ms)**, luminescent fluid **(lf)**, muscle **(m)**.

In *Platytroctes apus* we note the presence of tube organs on the caudal peduncle which have a similar morphology to the postcleithral tube organ (Fig 1C). These organs are darkly pigmented, arranged in series, and number from two to five distinct darkly pigmented (i.e., black) tube-like structures on both the dorsal and ventral margins of the caudal peduncle depending on the specimen examined (n = 14, Figs 1C and 5, S1 Fig). Each caudal tube organ has an associated modified scale (ms) that is concavely curled and slightly elevated compared to adjacent scales (Figs 1C and 5). We note the presence of numerous luminescent-fluid cells (lf) in each of the caudal light organs, ranging in size from 14–26 μm (Fig 5A). We also observe smaller cells (*) in the walls of the caudal light organs, ranging in size from 4–8 μm (Fig 5B). We find that caudal tube organs are lacking entirely (i.e., they are not present on either the dorsal or ventral margins of the caudal peduncle) in all examined specimens of *P. mirus* (n = 17, S1 Fig), the only other currently recognized species in the genus *Platytroctes*, as well as in all other platytroctid species examined.

## Discussion

In this study we describe the general morphology and ultrastructure of the postcleithral tube organ in 10 of the 13 genera of Platytroctidae and provide the first detailed description of the caudal tube organs unique to *Platytroctes apus*. While it is widely accepted that Platytroctidae is monophyletic and recovered within the order Alepocephaliformes, our understanding of the phylogenetic relationships within Platytroctidae are limited to few studies, none of which are taxonomically comprehensive [20]. We find that the overall structure of the postcleithral tube organ is generally conserved across Platytroctidae, supporting the evolution of the postcleithral tube organ in the ancestor of all platytroctids. We note that a postcleithral tube organ is lacking in all other alepocephaliform species [32,36]. However, a few species-specific anatomical differences are noted and described in detail below.

We observe a dark pigment layer along the internal surface of the tube organ in all species investigated (Figs 3 and 4). This aligns with previous observations of the dark internal lining of the tube organ [35]. However, we find that this pigment layer is much thicker on the internal apical surface of tube organ lumen, closer to the external environment (Figs 3 and 4). We also observe that some species exhibit a thicker pigment layer than others. *Holtbyrnia melanocephala* (Fig 4D) has an extremely thick pigment layer in the dermis lining the postcleithral tube, whereas the pigment layer is thin and reduced in *Mirorictus taningi* (Fig 3A). While darker pigments are generally associated with greater depths in fish [37], pigment layer thickness of the tube organ is likely not related to habitat depth in Platytroctidae, as mesopelagic genera (e.g., *Holtbyrnia*, *Searsia*) [32] have pigment layers of varying thickness. Instead, thicker pigment layers in the postcleithral tube organ are observed in species with darker overall body pigmentation (e.g., *H. melanocephala,* Fig 4D). A similar pigment layer is seen in the light organ of one of the only other vertebrate species known to emit bioluminescent fluid, the American Pocket Shark (*Mollisquama* *mississippiensis*), which is hypothesized to expel bioluminescent fluid via the movement of the closely associated pectoral fin [19]. This layer of pigmentation likely functions to prevent light from the bioluminescent fluid from being visible to the external environment while it is within the tube organ prior to secretion.

A modified scale is found to support the tube organ in all but one species that we examined (Figs 1B, 3A-B, and 4). The absence of a modified supporting scale in *Holtbyrnia macrops* may be due to accidental removal during collection via abrasive forces, given that our specimen of *H. macrops* is relatively small (21.9 mm SL) and lacks any observable body scales. The modified scale is hypothesized to provide structure and stability to the tube organ, particularly distally, near its opening to the environment (Fig 2) [34]. At the terminal, external tip of the tube, the modified scale is folded into a funnel-like shape (Fig 1B), which may function to direct the luminescent fluid during expulsion [20]. For example, in live *Searsia koefoedi*, applying gentle external pressure to the shoulder region resulted in a jet of luminescent fluid being ejected from the tube organ up to 25 cm in the air [38]. However, recently deceased specimens of *S. koefoedi* in air responded to the same stimulus by slowly releasing the luminescent fluid onto the flank of the organism [38], akin to observations of luminescent secretions in recently deceased *Sagamichthys schnakenbecki* [20]. This may suggest a mechanism of muscular control of the expulsion of luminescent fluid, perhaps by contraction of the adjacent hypaxial muscle layer (Figs 3 and 4).

### Tube organ cell types/comparison to Nicol [22] (1958)

We find similar cell types within the postcleithral tube organ to those reported by Nicol [22], the only histological study of the postcleithral tube organ in platytroctids to date. Despite investigating only one species, *Sagamichthys schnakenbecki*, Nicol [22] found three potentially luminescent cell types associated with the tube organ: red-stained spherical cells in the lumen (8–40 μm), violet-stained cells in the walls and lumen of the tube (5um), and blue-stained cells in the lumen (6–8 μm). Our histological observations reveal a large number of luminescent-fluid cells (lf) of similar size (8–32 μm) to Nicol’s “red cells” [22] in the lumen of the tube organ in many species (Figs 3 and 4, Table 2, S1 Table). We also find smaller cells (2–7 μm) that are stained dark violet, and are often associated with the pigment layer (Fig 3C and D, Table 2, S1 Table). These cells (*) are likely the “violet cells” observed by Nicol [22]. We also observe slightly larger cells that have an unstained or very light blue cytoplasm with a dark violet nucleus, which are likely the “blue cells” (bc) reported by Nicol [22] (Fig 3C) that are noticeably rounder and contain more cytoplasm. Whereas all three cell types were not observed in all specimens examined (Table 2), that does not necessarily mean those specimens lack those cell types. For example, a lack of luminescent fluid cells could mean the individual expelled the fluid during capture. Further, it was difficult to make conclusions on either cell anatomy or cell presence in smaller more delicate specimens that did not produce as high-quality histological samples as compared to larger, more well-preserved specimens. Thus, we focus on cell types that were present in the majority of specimens until additional specimens become available for dissection and histological examination.

Nicol [22] hypothesized that the “violet cells” were generative tissue that pass into the lumen of the gland and transition to the “blue cells”, which develop into larger, luminous “red cells” [i.e., luminescent fluid (lf) cells]. However, Nicol [22] was hesitant regarding this hypothesis, as no such exocrine luminous secretions are known to be dermally derived [19,39]. The platytroctid postcleithral tube organ lacks a prominent epithelial layer with secretory cells (e.g., goblet cells) or an apical layer from a stratified epithelium that could be the source of the cellular luminescent discharge. It is possible that the small violet cells (*) (Fig 3C and D) we observe could be clumps of epithelium. However, we are uncertain whether these rarely observed cells represent true epithelial cells, or if they are even present in a sufficient quantity to potentially support the number of luminescent-fluid cells observed. Interestingly, we do find a thin layer of epithelium associated with the numerous blood vessels that are present in the collagen-rich connective tissue near the walls of the tube organ (Fig 3C). We usually find red blood cells (5–12 μm) within these vessels as well (Fig 3C). Thus, whereas our observed cell types are consistent with Nicol’s [22] observations, the hypothesized mechanism of a dermally derived exocrine gland is unlikely. Instead, it seems more plausible that the luminescent-fluid cells, and the potential precursor cell types, originate outside the tube organ, perhaps in the proximate hematopoietic head kidney. However, further studies are needed to determine whether a direct connection exists between the postcleithral tube organ and head kidney.

### Caudal peduncle tube organs

In a single species, *Platytroctes apus*, we observe the presence of tube-like organs on the caudal peduncle that are of a similar morphology to the postcleithral tube organ. These organs are arranged in a median series and number from two to five darkly pigmented distinct tube-like organs on both the dorsal and ventral margins of the caudal peduncle depending on the specimen examined (Figs 1C and 5). Based on our histological analyses, we find that the caudal tube organs in *P. apus* are structurally similar to the postcleithral tube organ common to all members of Platytroctidae. Each tube-like caudal organ has a modified scale supporting the external opening of the tube organ, a darkly pigmented lining, and all three cell types of the developing luminescent fluid as described above (Fig 5). However, the modified supporting scale in the caudal tube organs is folded into a U-shape and oriented away from the body (Figs 1C and 5), unlike the modified supporting scale of the postcleithral tube organ, which folds tightly inward towards the body (Fig 1B). As a result, the caudal tube organs lack a distinct tube tip, and instead resemble more of a terminal scoop. Based on the overall similar anatomy of the postcleithral and caudal tube organs, and the presence of the same luminescent-fluid cells in both types of organ, we hypothesize that the caudal tube organs of *P. apus* can emit bioluminescent fluid and that they function similarly to the postcleithral tube organ [40]. *Platytroctes mirus*, the only other currently recognized species in the genus *Platytroctes*, lacks caudal tube-like organs entirely, as we did not observe them on either the dorsal or ventral margins of the caudal peduncle.

## Conclusion

In this study we investigate the structure of the postcleithral tube organ across 14 species of platytroctids, representing 10 of 13 total genera (Table 1; Figs 1B, 2–4). We provide the first histological analysis and detailed description of the unique caudal tube organs found only in *Platytroctes apus*, showing that these organs are similar in structure to the postcleithral tube organ (Figs 1C, 5). We find that the postcleithral tube organ is a highly vascularized sac-like structure with a modified supporting scale, an internal apically distributed layer of dark pigment lining the tube lumen, and with no continuous epithelial layer lining the lumen of the organ (Figs 2–4). We also observe three cell types in agreement with the original findings of Nicol [22] in both the postcleithral tube organ, as well as the caudal tube organs of *P. apus*: clumps of smaller cells (*) in the tube organ wall, poorly stained “blue cells” in the lumen, and numerous larger luminescent-fluid cells also in the lumen (Fig 3).

Whereas we agree with Nicol’s [21] observations regarding cell types present in the tube organs, we propose an alternative hypothesis regarding the origin of the luminescent fluid in the postcleithral tube organ. Due to the lack of a layer of epithelium and a high degree of vascularization, the bioluminescent fluid in the platytroctid postcleithral tube organ may originate via hematopoietic processes in the proximate head kidney. Although we have not yet been able to determine whether there is a direct connection between the postcleithral tube organ and the head kidney, we note that the head kidney and lumen of the postcleithral tube organ are in close proximity. In order to test this hypothesis, further studies are needed to understand the metabolic processes leading to the production of functional luminescent cells found in the platytroctid postcleithral tube organ and determine whether a direct connection exists between the postcleithral tube organ and head kidney. Regardless, luminescent cell production appears to be the result of a unique mechanism in tubeshoulders unlike anything documented to date in teleost fishes.

## Supporting information

S1 Fig Specimens examined for presence of caudal tube organs in addition to specimens used for histological analyses listed in Table 1. (DOCX)

S2 Fig Longitudinal sections through the postcleithral tube organ. (A and B) *Barbantus curvifrons*, (C and D) *Holtbyrnia latifrons*, (E) *Holtbyrnia macrops*, (F) *Holtbyrnia melanocephala*, (G and H) *Maulisia microlepis*, (I) *Mentodus facilis*, (J) *Normichthys osperosus*, (K) *Normichthys yahganorum*, (L) *Sagamichthys abei*, (M and N) *Platytroctes mirus*, (O) *Searsia koefoedi*, (P) *Sagamichthys abei*. Anterior is to the left in all figure panels, such that the posteriorly-directed terminal opening of the tube organ is to the right. Abbreviations: scale (s), dermis (d), tube organ (t), pigment layer (pl), modified scale (ms), luminescent fluid (lf), potential developing luminescent-fluid cells (*), muscle (m), nucleus (n), blue cells (bc), red blood cells (rb), blood vessel (v), epithelium (ep).
(TIF)

S1 Table Measurements of various cell types in Platytroctidae specimens. If a measurement for a particular cell type is missing for a given species, that cell type was not observed in the specimens examined in this study.
(XLSX)
