# Supplementary material for: A shot in the dark: Comparative morphology of the bioluminescent tube organs in tubeshoulders (Platytroctidae)
Source: PLoS One. 2026 Jan 14;21(1):e0332016. doi: 10.1371/journal.pone.0332016 (PMC12803472; doi:10.1371/journal.pone.0332016)
Supplement: S1 Fig — (DOCX) [file pone.0332016.s001.docx]

**Supplementary File 1**

Specimens examined for presence of caudal tube organs in addition to specimens used for histological analyses listed in Table 1:

*Platytroctes mirus*: MCZ 138114, n=3, 49.4-115.8 mm SL, Arabian Sea, Somalia.

*Platytroctes mirus*: MCZ 138115: n=1, 108.0 mm SL, Arabian Sea, Oman.

*Platytroctes mirus*: MCZ 138711, n=2, 96.4-100.4 mm SL, Arabian Sea.

*Platytroctes mirus*: MCZ 138712, n=3, 74.8-104.9 mm SL, Arabian Sea, Oman.

*Platytroctes mirus*: MCZ 138717, n=2, 60.8-83.2 mm SL, Arabian Sea.

*Platytroctes mirus*: MCZ 138719, n=2, 47.2-76.5 mm SL, Arabian Sea, Oman.

*Platytroctes mirus*: MCZ 140530, n=2, 109.6-110.0 mm SL, Arabian Sea, Yemen.

*Platytroctes mirus*: MCZ 144571, n=1, 97.9 mm SL, Arabian Sea, Yemen.

*Platytroctes mirus*: USNM 200471, n=1, 50.2 mm SL, Indian Ocean.

*Platytroctes apus*: SIO 55-244, n=1, 136.4 mm SL, E. Tropical Pacific Ocean.

*Platytroctes apus*: SIO 77-54, n=1, 128.0 mm SL, Indo-W. Pacific Ocean.

*Platytroctes apus*: USNM 201649, n=1, 138.4 mm SL, S.W. Gulf of Mexico.

*Platytroctes apus*: USNM 206871, n=3, 111.2, 119.1, 153.6 mm SL, E. North Atlantic Ocean.

*Platytroctes apus*: USNM 206893, n=2, 120.8-132.4 mm SL, Madeira Islands.

*Platytroctes apus*: USNM 206894, n=2, 78.3-110.9 mm SL, Cape Verde.

*Platytroctes apus*: USNM 240126, n=2, 88.2 mm SL, Bermuda.

*Platytroctes apus*: MCZ 163133, n=1, 95.0 mm SL, N.W. Atlantic Ocean, United States.

*Platytroctes apus*: MCZ 45305, n=1, 65.5 mm SL, W. Indian Ocean, Mauritius.

*Normichthys yahganorum*: SIO 61-37, n=1, 50.5 mm SL, Indian Ocean.
